# Supplementary material for: Few-femtosecond time-resolved study of the UV-induced dissociative dynamics of iodomethane
Source: Nat Commun. 2024 Oct 25;15:9196. doi: 10.1038/s41467-024-53183-8 (PMC11511850; doi:10.1038/s41467-024-53183-8)
Supplement: Supplementary file 1 — Supplementary Information [file 41467_2024_53183_MOESM1_ESM.pdf]

Supplementary Information:

“Few-femtosecond time-resolved study of the UV-induced dissociative dynamics of iodomethane”

Lorenzo Colaizzi, Sergey Ryabchuk, Erik P. Månsson, Krishna Saraswathula, Vincent Wanie, Andrea Trabattoni, Jesús González-Vázquez, Fernando Martín and Francesca Calegari

Supplementary Note 1: Laser pulses properties

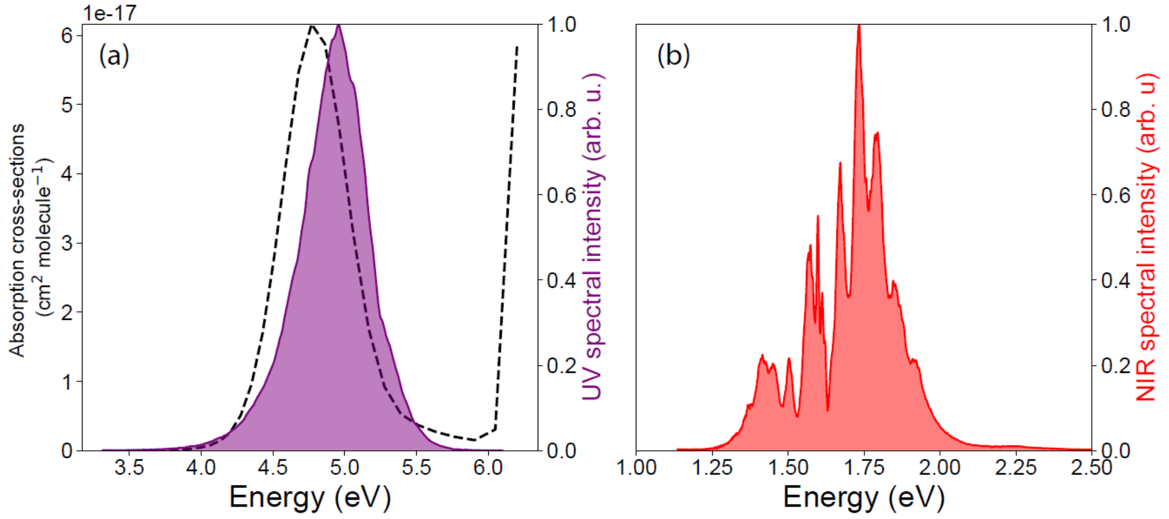

Supplementary Figure 1. Pump and probe spectra. (a) Pump spectrum (purple filled solid line) and methyl-iodide cross-section (dashed line) based on data reported in Table 1 of Supplementary Ref.[1]. (b) Probe spectrum (red filled solid line).

The spectra of the UV pump and the NIR probe spectra described in the Methods section are shown in Supplementary Fig. 1.

The UV pulse duration has been estimated in situ through two-color cross-correlation in Krypton. The gas is injected through the same sample delivery system described for CH_3I in the Methods section. Due to an ionization energy of 13.99 eV, a minimum of two UV + three NIR photons are needed to ionize the atom. The energy and angular distribution of the photoelectrons are measured through a Velocity Map Imaging (VMI) spectrometer[2] which is integrated in our Time-of-Flight spectrometer[3]. The VMI images have been acquired as a function of the NIR delay averaging each point over 3×10^4 laser shots. The images have been Abel inverted[4] and then angularly and energy integrated in an energy range below 1 eV. The result is shown in Supplementary Fig. 2. The W_{CC} width of the Gaussian at time zero is related to the W_{UV} and W_{NIR} full width at half maximum (FWHM) durations through the following cross-correlation expression:

$$W_{\mathrm{cc}} = \sqrt{W_{\mathrm{UV}}^2/n_{\mathrm{UV}} + W_{\mathrm{NIR}}^2/n_{\mathrm{NIR}} + W_{\mathrm{nc}}^2} \quad (1)$$

where n_{UV} and n_{NIR} are the number of UV and NIR photons involved in the ionization and W_{nc} is an additional term that accounts for the non-collinear geometry used in the experiment. Considering an angle of about 0.6° between the beams, we obtain a W_{nc} of 1.2 fs (for the three-photon probing case). The NIR pulse can be characterized independently with an SHG FROG setup, resulting in W_{NIR} = (5.6 ± 0.2) fs. Therefore, we estimate a UV FWHM duration W_{UV} = 4.2 ± 0.3 fs. The standard deviation is obtained by propagating the standard deviation of the fit through Supplementary Equation (1).

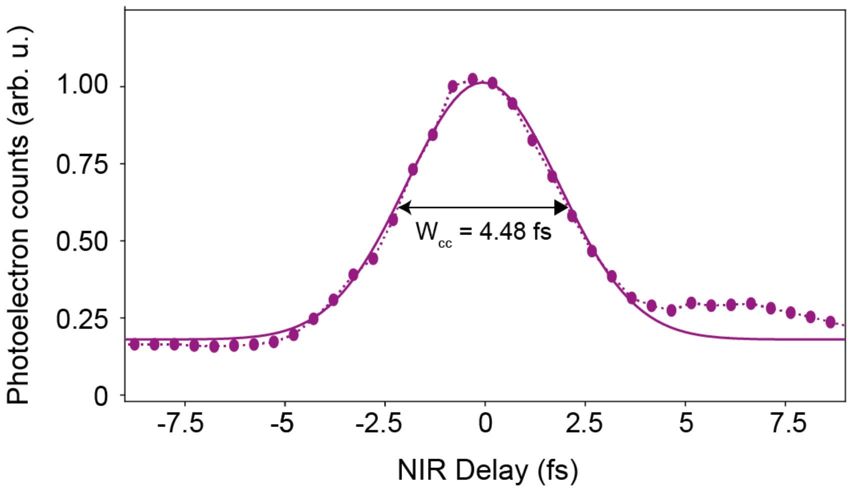

Supplementary Figure 2. Cross-correlation trace obtained by photoionizing Krypton gas. The trace is obtained using a VMI spectrometer and selecting photoelectrons with kinetic energy below 1 eV, which results from 2 UV + 3 NIR interaction. The solid line represents the Gaussian fit.

From the characterized FWHM of the UV (W_{UV}) and NIR (W_{NIR}) pulses and the number of absorbed NIR-photons in the probing step (n_{NIR}), the experimental time resolution (FWHM of the Gaussian instrument response function) can be estimated using Supplementary Equation (1) as 5.1 ± 0.3 fs. The small variation with n_{NIR} justifies the fitting of all the experimental signals with a common time-resolution parameter.

Supplementary Note 2: Mass spectra details

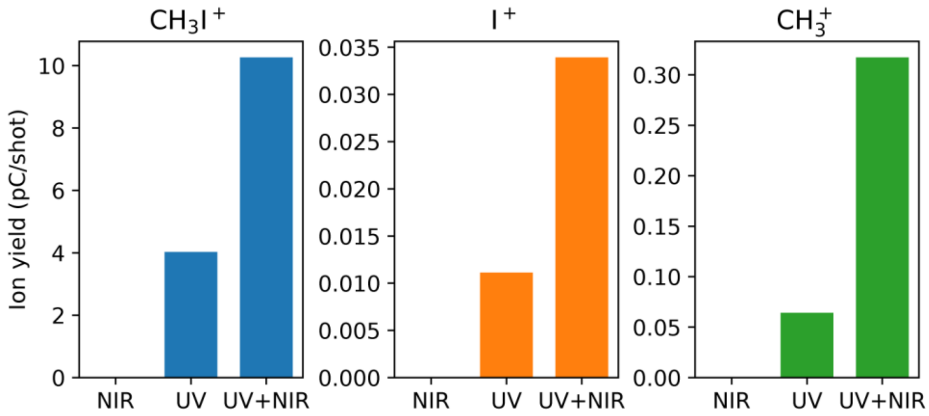

Supplementary Figure 3. Typical mass spectra. They were obtained for the cases of NIR-only, UV-only and UV+NIR pulses and for all the three ions detected in the experiment.

Typical mass spectra obtained for the cases of NIR-only, UV-only and UV+NIR pulses are shown in Supplementary Fig. 3. The NIR-only signal is negligible, while the UV-only spectra exhibit background signals due to two- and three-UV-photon transitions, which were also observed in previous works with similar pump intensities.[5,6] We have performed measurements at lower UV intensities to check that the observed time-dependent signals are reproducible and, therefore, mostly related to the single UV photon transition.

Supplementary Note 3: Extended time-dependent fragment signals

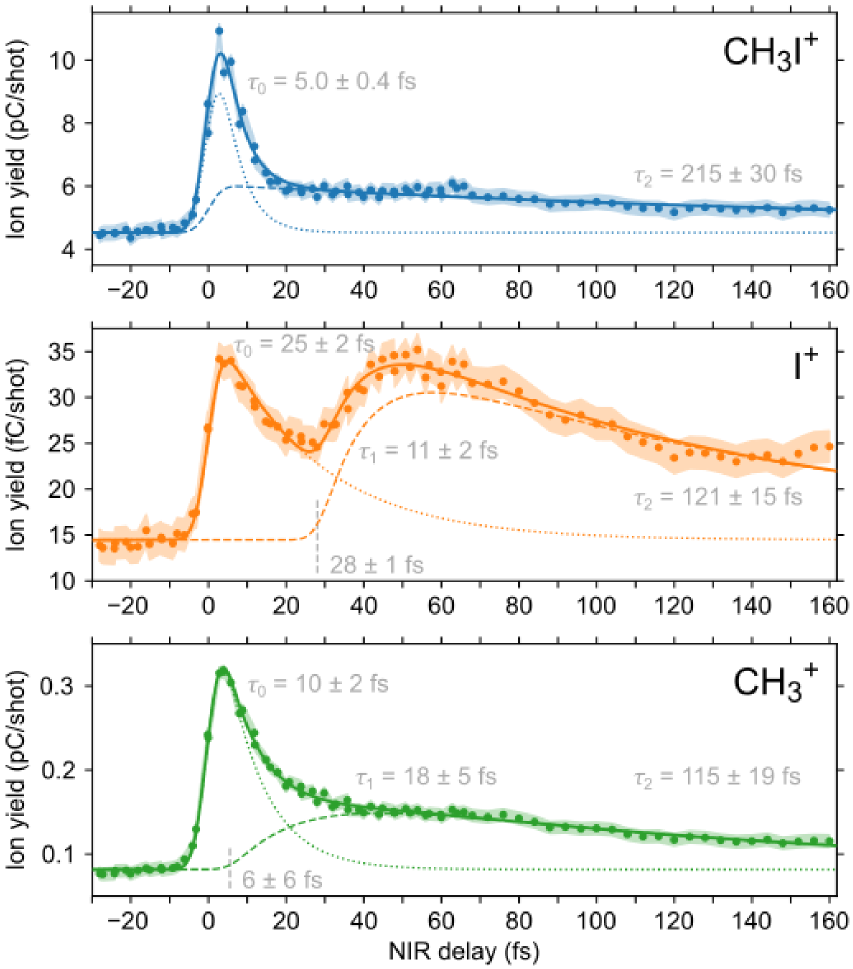

Supplementary Figure 4. Extended version of Figure 2. The experimental data and fitted curve model for the entire available delay range.

In Supplementary Fig. 4, we report the full dataset obtained by merging a scan performed with 3 fs steps up to 70 fs and a scan performed soon thereafter with 4 fs steps up to 160 fs. Their delay-axes were calibrated using initial single-scan fits using the same curve model as described in Supplementary Note 4. In each pump–probe scan, we acquired 6×10^4 laser shots per step forwards and again backwards to produce an average data point.

Supplementary Note 4: Fitting procedure

We use a global curve-fitting model consisting of three terms: a rapidly decaying peak, a long-lived contribution, and a constant baseline term. These models are characterized by the following terms: the rapidly decaying peak starts at $t = 0$ and is characterized by an exponential lifetime τ_0. The long-lived contribution is rising for $t > t_d$, where t_d represents a finite delay (time offset) with respect to $t = 0$ and a rising expressed by $(1 - \exp((t - t_d)/\tau_1)$ and finally a decaying term with lifetime τ_2. These terms are convoluted with a Gaussian function representing the experimental time resolution with a common FWHM parameter W.

The two exponential contributions have been fitted using the analytical expression used in Supplementary Ref.[7,8]. Similar curve models with time offsets, representing isomerization time or wave packet motion, have, for instance, been used in Supplementary Ref.[9–11]. The coefficients A_0 and A_1 denote the heights of the short and long-lived terms, respectively. For the parent ion, the second term was constrained to start abruptly and without delay, based on the Rydberg-like assignment, while a time shift of t_d was allowed in the second term for the fragment ions.

Ion	A_1/A_0	**baseline**	τ_0 **(fs)**	t_d **(fs)**	τ_1 **(fs)**	τ_2 **(fs)**
CH_3I^+	15%	4.53 ± 0.04	5.0 ± 0.4	0 (fixed)	0 (fixed)	215 ± 30
I^+	83%	(144 ± 3) ×10^{-4}	25 ± 2	28 ± 1	11 ± 2	121 ± 15
CH_3^+	24%	(81 ± 1) ×10^{-3}	10 ± 2	6 ± 6	18 ± 5	115 ± 19

Supplementary Table 1. Main results from the curve model fitted to the experimental data. The shared Gaussian FWHM parameter was fitted to *W* = 6.7 ± 0.3 fs.

Supplementary Note 5: Numerical calculation details

Visualization of the dissociative trajectories in the neutral molecule

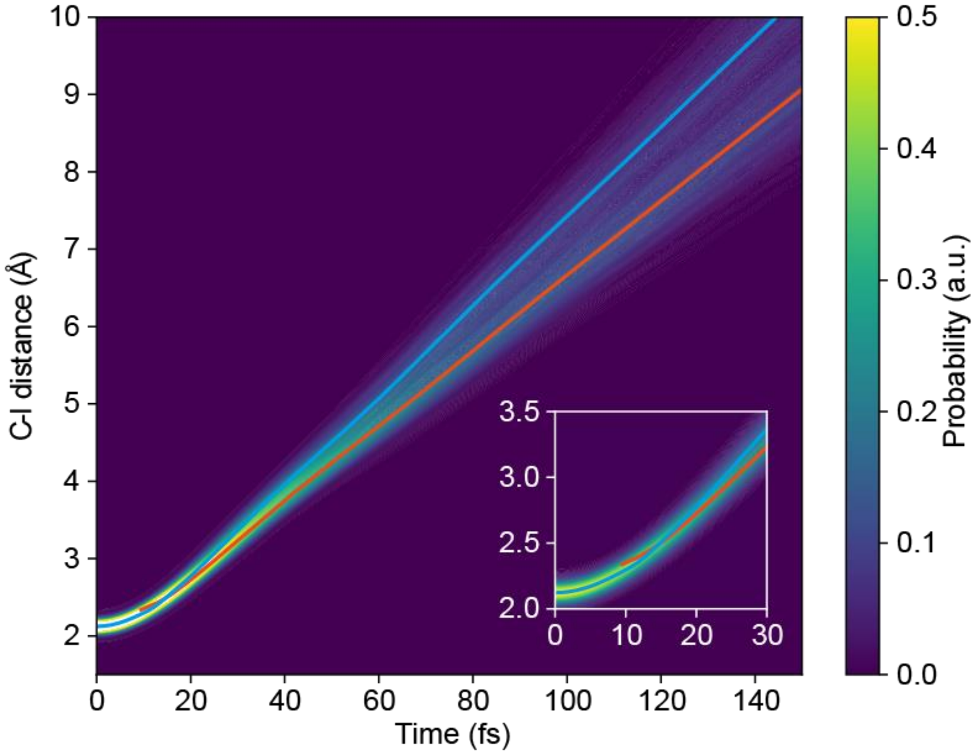

Supplementary Figure 5. **Weighted convolution of the C-I distance for all the trajectories as a function of time.** The convolution was done with a Gaussian function of 0.1 Angstroms FWHM. On top of the convoluted data, the weighted average distance when including the trajectories in the first 8 (cyan) and the last 4 (red) electronic states is also included. The inset focuses on the first 30 fs, where the bifurcation between the two channels I (cyan) and I* (red) can be observed.

Supplementary Fig. 5 shows the evolution of the average C-I distance for two sets of trajectories. In the first set, the average has been performed by considering the electronic states leading to dissociation into I (the 8 lowest states) and, in the second set, by considering the states leading to dissociation into I* (the 4 upper states). One can see two different evolutions: the faster one corresponds to dissociation into the I channels, whereas the slower one to dissociation into the I* channels. In the case of I*, a clear change of the slope at around 40 fs is observed (when a change of the I^+ channel is also observed experimentally). At short times, the bifurcation of the two channels, related to the passage through the conical intersection, is quite apparent at around 14.5 fs. This value is very similar to those obtained in refs. 13 (15 fs) and 16 (13 fs), but it is higher than that directly obtained from the energy difference between the states (10.5 fs). This 4-fs difference (between 10.5 fs and 14.5 fs) can be explained as the time required for the ensemble of trajectories to experience the CI-induced gradient and separate.

Derivation of n-photon theoretical curves

To obtain the curve related to the absorption of n photons, a selection of which is shown in Figure 3 (d–f) in the Results section of the manuscript, the trajectory count in the energy region between $n-1$ and n NIR photon energies has been summed and reported in Supplementary Fig. 6. Physically, this corresponds to assuming an electron spectrum with a uniform distribution of kinetic energies from $n-1$ to n NIR photon energy and that all ionization cross-sections are otherwise equal. In Supplementary Fig. 7, a similar sum has been performed by selecting only specific trajectories. The curves in Supplementary Fig. 7 panel (a-c) are obtained considering only trajectories ending in the $I(^2P_{3/2})$ channel and indicated by I_g. The curves in Supplementary Fig. 7 panel (d-f) represent only the trajectories in which the wave packet stays on the $^3Q_0$ and are correlated to the I* $(^2P_{1/2})$ channel, therefore indicated with the label I_s. The latter subset represents the trajectories as if no crossing occurred at the conical intersection and is also reported for the ion fragments in Figure 3 (e-f). The sum of the two subsets produced the total simulated trajectories reported in Supplementary Fig. 6.

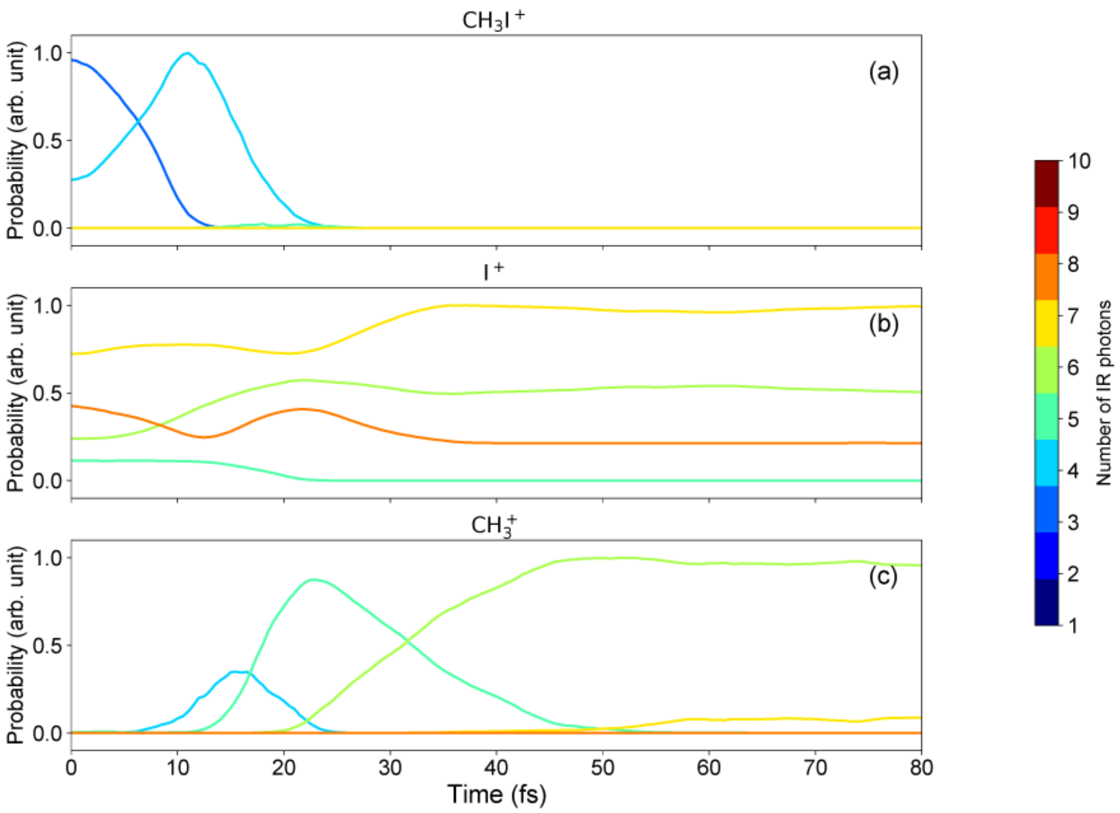

Supplementary Figure 6. Readouts from Figure 3 (a–c) as at the specified number of NIR photons for each ion (a-c). The curves are shown normalized to the highest value for each ion. A subset of these curves is shown also in Figure 3 (d–f).

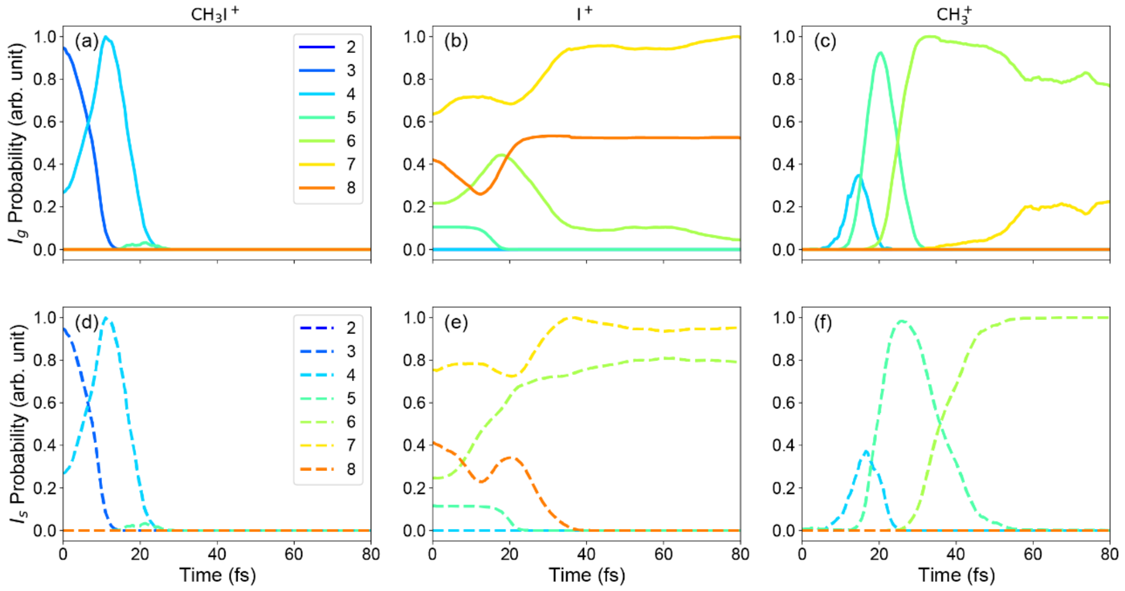

Supplementary Figure 7. The effect of the crossing on the trajectory leads to specific ion production at the number of NIR photons specified by the label. The curves are shown normalized to the highest value for each ion. Panel (a-c) shows the curves obtained when the dynamics end on the ground state of iodine I ($^2P_{3/2}$) (I_g). Panel (d-f) instead, results in the trajectory correlated with iodine in the excited state I* ($^2P_{1/2}$).

Relative contribution of the theoretically extracted probabilities

Although our theoretical model does not include information concerning the multiphoton cross-sections of the probing step, we could still use the set of theoretically retrieved curves (shown in Supplementary Fig. 6) to address their relative contribution to the experimental signals for each ion. First, in order to consider our finite time resolution, we convoluted each of the normalized curves with the instrumental response function reported in Supplementary Note 1. Subsequently, we used these curves as a basis set to decompose the experimental signal. Each experimental curve has been normalized to its maximum value, and the baseline extracted for negative delays has been subtracted. The normalized ion yield can thus be represented by a linear combination of the n-photon theoretical curves, where the A_n coefficients are obtained through a best fit routine. Each of these coefficients represents the relative contribution of each curve to the ion yield. The values obtained for each coefficient are reported in Supplementary Table 2.

Parameters	CH_3I^+	I^+	CH_3^+
A_3	0.88 ± 0.01	0 (fixed)	0 (fixed)
A_4	0.04 ± 0.01	0 (fixed)	0 (fixed)
A_5	0 (fixed)	0.33 ± 0.07	0.31 ± 0.02
A_6	0 (fixed)	0.00 ± 0.05	0.30 ± 0.01
A_7	0 (fixed)	0.63 ± 0.14	0.02 ± 0.02
A_8	0 (fixed)	0.00 ± 0.12	0 (fixed)

Supplementary Table 2. Coefficients were obtained by fitting the set of normalized curves from Supplementary Fig. 6 to the experimental data normalized to the maximum of each ion yield. A_n represents the relative contribution of the corresponding n-photon curve from Supplementary Fig. 6, along with the standard error resulting from the fitting procedure.

In Supplementary Fig. 8, we report the fitting curves as solid lines on top of the experimental data. The individual n-photon theoretical curves weighted by their relative coefficients extracted from the fit are reported in the same figure as colored solid lines.

Due to the rapid decay of the parent ion, the fit for this ion has been performed only up to 10 fs. Note that, for the parent ion, the three-photon transition is the dominant contribution to the signal, with only a smaller contribution from the four-photon curve. When considering the iodine cation, many more curves can contribute. However, in this case, only the coefficients A_5 and A_7 significantly differ from zero. Therefore, this fitting procedure confirms our previous conclusion that the six-photon transition only marginally contributes to the iodine signal. Finally, for the methyl fragment, only the coefficients A_5 and A_6 contribute to the long-lived component observed in the experimental data, once more corroborating our previous interpretation.

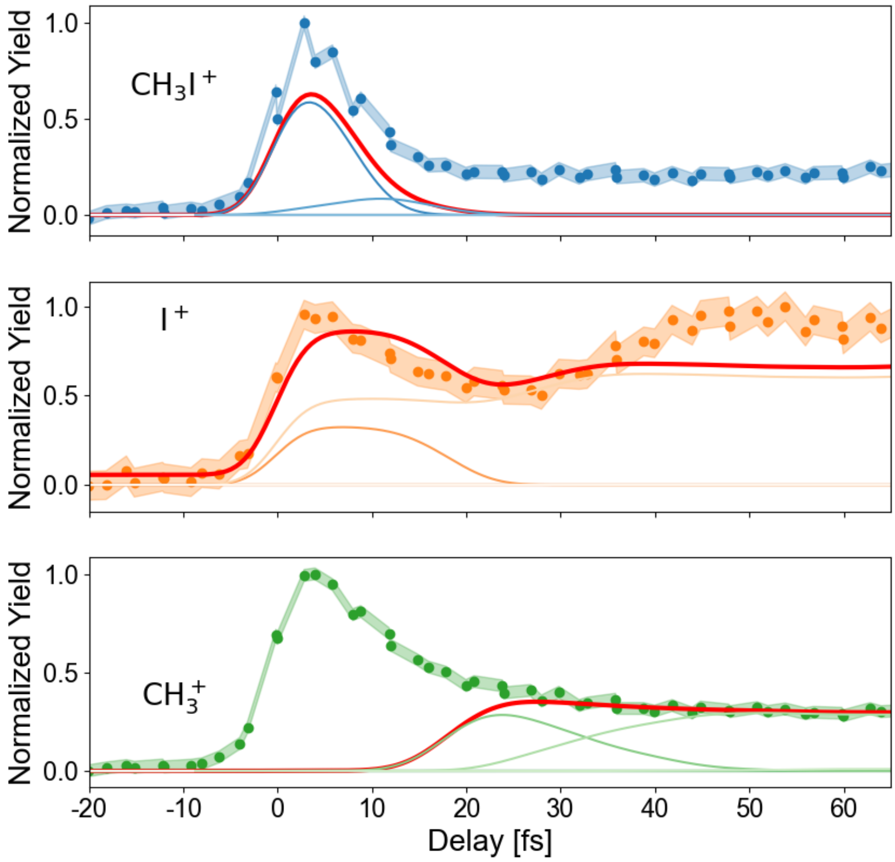

Supplementary Figure 8. Best fitting curves (red solid lines) obtained as a linear combination of the theoretical curves from Supplementary Fig. 6. Dots indicate experimental data points (normalized to the maximum value for each ion), while shaded areas indicate the standard deviation. The constant baseline for negative delays has been subtracted. The individual *n*-photon theoretical curves are weighted by their relative coefficients extracted from the fit and are also reported here as colored solid thin lines.

Time-Dependent Schrödinger Equation simulations

To better understand the multiphoton ionization process induced by the probe pulse, we have calculated the ionization probabilities to the different electronic states of the cation as a function of time delay by solving the time-dependent Schrödinger equation (TDSE) on a basis of molecular electronic eigenstates. For the neutral molecule and the corresponding cation, the energies and electronic states were obtained by using the geometries resulting from the calculated trajectories at every time step. Spin-orbit splittings, dipole couplings and Dyson orbitals for all these states were also obtained at every time step, in this case at the perturbated modified CASSCF level. The NIR-induced dipole couplings between the bound electronic states of the neutral molecule and between the electronic states of the molecular cation, respectively, were explicitly calculated and included for all states shown in Figure 1 of the Introduction section at each time delay. The transition matrix elements between the states of the neutral molecule and the cation were approximately represented by the corresponding Dyson norms. The latter were rescaled with a factor of 100 to account for the multiphoton nature of the transition and to obtain a non-negligible value of the ionization probability.

Electronic continuum states up to 2 eV above the various ionization thresholds were mimicked by replicating 20 times the corresponding states of the cation (displacing the cationic states with the corresponding kinetic energy). In this way, the model included a total of 692 coupled states at each time delay. We note that by restricting the number of bound states of the neutral molecule to those shown in Figure 1 of the main text, we are ignoring the possible effect of the Rydberg states in the multiphoton ionization process. However, since dipole transition moments associated with these states rapidly decrease as they approach the ionization limit, we do not expect them to play a significant role. We also note that, although some of the dissociation products of the molecular cation (mainly ICH_2^+) cannot be retrieved by using this approach[12], the procedure gives a reasonable representation of the Frank-Condon energies and the dissociation limits for the CH_3^+ and I^+ cations[13].

In the calculations, a sine square laser pulse of FWHM duration 5 fs, central frequency 1.7 eV and intensity 6.47×10^{11} W/cm^2 was used to mimic the experimental one. The laser-molecule interaction was described within the length gauge. At each time delay, the initial wave function was that in the active potential of the trajectory. This wave function was then propagated during 10 fs with a time step of 0.01 fs. The calculated transition probabilities were assigned to the different fragmentation channels by using Eq. 1 in the Results section and the same energetic criteria.

It is worth mentioning that in this description of multiphoton ionization by the probe pulse, the electronic TDSE must be solved for each nuclear geometry reached in a given trajectory (150) and for all the trajectories (1,000), which amounts to 150,000 TDSE calculations. This provides the multiphoton ionization probabilities to the different states of the cation for all considered nuclear geometries. However, these probabilities do not tell us if the molecule will subsequently dissociate or not. To know this, one has to consider that, at the instant of ionization by the probe pulse, the nuclei have already acquired a certain amount of kinetic energy E_{kin}. This quantity is easily extracted from the calculated nuclear trajectories. When the sum $E_{kin} + E_{pot}$ is larger than the dissociation energy D_i in a given state of the cation (Eq. 1 of the main text), multiphoton ionization will be followed by dissociation leading to fragment i. Otherwise, the molecular cation will not dissociate.

Supplementary Fig. 9 shows the results of these calculations for the three fragmentation channels considered in this work.

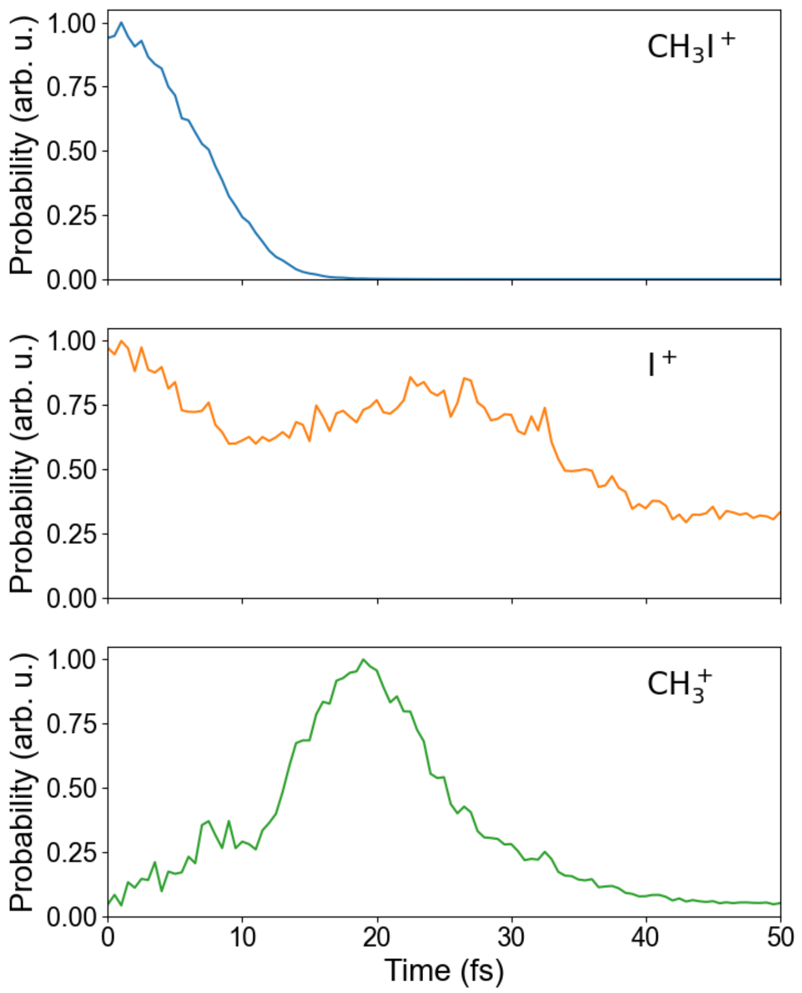

Supplementary Figure 9. Results of the TDSE simulation. Each curve probability has been normalized to a maximum of 1.

Overall, the results confirm the behavior discussed in the main text and support the analysis based on the much simpler model described there. Following excitation, the parent undergoes a rapid decay, and the iodine fragmentation probability follows a non-monotonous behavior. Additionally, the methyl fragment is shown to rise during the first 15 fs and decay to zero at around 30 fs. The rising of the signal agrees with the behavior reported in Fig. 2 of the Results section for the four-photon and the five-photon contribution, while the former six-photon contribution seems to be missing. This fast decay does not agree with what is observed experimentally, however we can safely assume that our interpretation of the mechanisms occurring at shorter time delays (NIR-induced stabilization and passage through the CI), where the agreement with theory is satisfactory, remains unaffected. As reported in the main manuscript for the simpler model, the peak in the experimental signal for the methyl ion at zero-time delay is due to an internal conversion mechanism in the molecular cation, which is not included in the TDSE calculation and therefore is missing in the curve reported in the bottom panel of Supplementary Fig. 9.

Both the TDSE simulations and the simpler model described in the main text predict that there are more trajectories leading to I^+ than to CH_3^+ at long times, in contrast to the experimental finding. The reason for this behavior is that in both types of simulations the number of states dissociating into I^+ is considerably larger than the number of states dissociating into CH_3^+.

Supplementary References

1. Roehl, C. M., Burkholder, J. B., Moortgat, G. K., Ravishankara, A. R. & Crutzen, P. J. Temperature dependence of UV absorption cross sections and atmospheric implications of several alkyl iodides. *J. Geophys. Res.* **102**, 12819–12829 (1997).
2. Eppink, A. T. J. B. & Parker, D. H. Velocity map imaging of ions and electrons using electrostatic lenses: Application in photoelectron and photofragment ion imaging of molecular oxygen. *Review of Scientific Instruments* **68**, 3477–3484 (1997).
3. Månsson, E. P. *et al.* High-resolution mass spectrometry and velocity map imaging for ultrafast electron dynamics in complex biomolecules. *EPJ Web Conf.* **205**, 03007 (2019).
4. Gibson, S. *et al.* PyAbel/PyAbel: v0.8.4. Zenodo https://doi.org/10.5281/zenodo.4690660 (2021).
5. Chang, K. F. *et al.* Mapping wave packet bifurcation at a conical intersection in CH3I by attosecond XUV transient absorption spectroscopy. *J. Chem. Phys.* **154**, 234301 (2021).
6. Baumann, A., Rompotis, D., Schepp, O., Wieland, M. & Drescher, M. Time-Resolved Dissociation Dynamics of Iodomethane Resulting from Rydberg and Valence Excitation. *J. Phys. Chem. A* **122**, 4779–4784 (2018).
7. Lacoursière, J., Meyer, M., Nahon, L., Morin, P. & Larzillière, M. Time-resolved pump-probe photoelectron spectroscopy of helium using a mode-locked laser synchronized with synchrotron radiation pulses. *Nuclear Instruments and Methods in Physics Research Section A: Accelerators, Spectrometers, Detectors and Associated Equipment* **351**, 545–553 (1994).
8. Månsson, E. P. *et al.* Real-time observation of a correlation-driven sub 3 fs charge migration in ionised adenine. *Commun Chem* **4**, 1–7 (2021).

9. M. Warne, E. *et al.* Photodissociation dynamics of methyl iodide probed using femtosecond extreme ultraviolet photoelectron spectroscopy. *Physical Chemistry Chemical Physics* **22**, 25695–25703 (2020).
10. Murillo-Sánchez, M. L. *et al.* Femtosecond XUV–IR induced photodynamics in the methyl iodide cation. *New J. Phys.* **23**, 073023 (2021).
11. Tilborg, J. van *et al.* Femtosecond isomerization dynamics in the ethylene cation measured in an EUV-pump NIR-probe configuration. *J. Phys. B: At. Mol. Opt. Phys.* **42**, 081002 (2009).
12. González-Vázquez, J., A. García, G., V. Chicharro, D., Bañares, L. & Marggi Poullain, S. Evidencing an elusive conical intersection in the dissociative photoionization of methyl iodide. *Chemical Science* **15**, 3203–3213 (2024).
13. Murillo-Sánchez, M. L., Zanchet, A., Marggi Poullain, S., González-Vázquez, J. & Bañares, L. Structural dynamics effects on the electronic predissociation of alkyl iodides. *Sci Rep* **10**, 6700 (2020).
