# Supplementary material for: Few-femtosecond time-resolved study of the UV-induced dissociative dynamics of iodomethane
Source: Nat Commun. 2024 Oct 25;15:9196. doi: 10.1038/s41467-024-53183-8 (PMC11511850; doi:10.1038/s41467-024-53183-8)
Supplement: Supplementary file 3 — Description of Additional Supplementary Files [file 41467_2024_53183_MOESM3_ESM.pdf]

**Description of Additional Supplementary Files**

File Name: Supplementary Data 1
Description: With this Supplementary Data we are providing the initial and final geometries and velocities of the run trajectories
